# In Vitro Antioxidant and Cancer Inhibitory Activity of a Colored Avocado Seed Extract

**DOI:** 10.1155/2019/6509421

**Published:** 2019-04-24

**Authors:** Deepti Dabas, Ryan J. Elias, Gregory R. Ziegler, Joshua D. Lambert

**Affiliations:** ^1^Department of Food Science, The Pennsylvania State University, University Park, PA 16802, USA; ^2^Center for Molecular Toxicology and Carcinogenesis, The Pennsylvania State University, University Park, PA 16802, USA

## Abstract

Avocado (*Persea americana*) seeds have been used traditionally for a number of health-related indications. Because of its high polyphenol content, we investigated the potential antioxidant and anticancer effects of a colored avocado seed extract (CASE). CASE exhibited an oxygen radical acceptance capacity value of 2012 ± 300 trolox equivalents/mg. CASE reduced lipid hydroperoxide formation in an oil-in-water emulsion (33% reduction at 500 *μ*g/mL). CASE dose-dependently reduced the viability of human breast (MCF7), lung (H1299), colon (HT29), and prostate (LNCaP) cancer cells* in vitro*. The half maximal inhibitory concentrations ranged from 19 to 132 *μ*g/mL after treatment for 48 h. CASE treatment downregulated the expression of cyclin D_1_ and E_2_ in LNCaP cells. This was associated with cell G_0_/G_1_ phase cycle arrest. CASE also dose-dependently induced apoptosis in LNCaP cells. CASE reduced nuclear translocation of nuclear factor *κ*B, a prosurvival signal. Further studies are needed to examine these effects in* in vivo* models.

## 1. Introduction

Avocado (*Persea americana*, Lauraceae) is an important tropical crop that is rich in unsaturated fatty acids, fiber, vitamins B and E, and other nutrients. The Hass avocado is the most important variety grown commercially. In 2016, Mexico, the world's largest grower, produced 1.9 million metric tons of Hass avocados, while the U.S. produced 172,630 metric tons (http://www.fao.org). The seed of the Hass avocado accounts for approximately 16% of the total weight of the avocado fruit and is considered a low-value waste product: it can be estimated then that the Mexican Hass avocado crop generated 304,000 metric tons of seed waste in 2016 [1].

Ethnopharmacological studies of the Aztec and Maya cultures have reported the use of decoctions of avocado seeds for the treatment of mycotic and parasitic infections, diabetes, inflammation, and gastrointestinal irregularity. Our previous review highlights multiple potential applications of avocado seeds including insecticidal, antimicrobial, antidiabetic, and blood pressure reducing effects [2].

Avocado seeds are rich in polyphenols and contain a large number of different classes of phytochemicals. The seed has higher polyphenol content and greater antioxidant activity than the pulp [3–5]. Wang* et al.* have reported the presence of catechin, epicatechin, and A- and B-type procyanidin (PAC) dimers and hexamers in the seed. The seeds have also been reported to contain phytosterols, triterpenes, fatty acids, furanoic acids, and abscisic acid [5]. Melgar* et al. *identified many polyphenolics from the hydroethanolic extract of seed including isohametin-glucouronide, catechin, epicatechin, trans-3-O-Caffeoylquinic acid, B-type PAC dimer and trimer, cis 3-O-caffeylquinic acid, and cis-3-p-coumaroylquinic acid [6]. These authors compared the polyphenolic content of seeds and peels and found the peels to have 3-fold higher polyphenolic content, but only around twice the antioxidant activity. The extracts also displayed bactericidal and fungicidal characteristics [6].

To date, a limited number of studies have investigated the cancer-related effects of avocado seeds. Treatment of MDA-MB-231 human breast cancer cells with a methanolic extract of avocado seed led to induction of apoptosis as measured by increased caspase-3, caspase-7, and poly (ADP-ribose) polymerase (PARP) cleavage and increased DNA laddering [7]. Abubakar, Achmadi, & Suparto (2017) isolated a triterpenoid fraction from an ethanolic extract of avocado seeds and studied its cytotoxic effects in MCF7 breast cells [8]. They found that the triterpenoid fraction and the whole extract had IC_50_ values of 80.1 *μ*g/mL and 99.7 *μ*g/mL, respectively. Kristanty, Suriawati, & Sulistiyo (2014) found that the cytotoxicity of aqueous and ethanolic extract of avocado seeds inhibited T47D breast cancer cell line with IC_50_ values of 560.2 *μ*g/mL and 107.2 *μ*g/mL, respectively [9].

We have previously reported that when the avocado seed is crushed, a stable orange color develops, and we have investigated the potential use of this colored avocado seed extract (CASE) as a food color additive. CASE contains high concentrations of polyphenols (219.4 ± 4.5 mg/g GAE) [1]. Among the compounds identified in CASE are perseitol, abscisic acid, epicatechin/catechin, PAC B2, and salidroside [10]. The principal colored compound in CASE has been identified as a novel glycosylated benzotropolone -containing polyphenol [10]. A large number of studies have demonstrated the potential cancer inhibitory activities of benzotropolone-containing natural products such as theaflavins from black tea [11]. Based on these previous studies on the cancer inhibitory activities of benzotropolones, the potential usefulness of a CASE as a food additive, and the general lack of studies on the cancer inhibitory activity of avocado seed extracts, an investigation of the potential cancer inhibitory activity of this new extract was warranted. We hypothesized that CASE would exhibit dose-dependent antioxidant and cancer inhibitory activity* in vitro*.

## 2. Materials and Methods

### 2.1. Reagents

Ripe avocados (*P. americana*, Hass variety) were sourced from local grocery stores and stored at 4°C until use. The antibodies against cleaved caspase 3, PARP, cyclin D_1_, cyclin E_2_, *β* actin, and nuclear factor (NF)*κ*B were purchased from Cell Signaling (Danvers, MA). The antibody against cyclin A was purchased from Santa Cruz Biotechnology (Santa Cruz, CA). Fluorescent-conjugated secondary antibodies were purchased from Li-Cor Biosciences Co. (Lincoln, NE). All other reagents were of the highest grade commercially available.

### 2.2. Preparation of CASE

CASE was prepared as previously described [1]. In brief, avocado seeds were separated from the fruit, washed, and peeled. Seeds were ground in 0.7 vol. of deionized (DI) water using a Waring Blender. The resulting paste (pH 6.4) was incubated at 24°C for 35 min. The colored paste was transferred to a beaker, an equal volume of methanol was added, and the mixture was sonicated for 20 min; an additional 2 vol. of methanol was added, and the mixture was centrifuged at 1200 × g for 10 min. Methanol was removed using a rotary evaporator and the water was removed by freeze-drying. Stock solutions (200 mg/mL) were prepared in dimethyl sulfoxide and stored at -80°C.

### 2.3. Oxygen Radical Absorbance Capacity (ORAC) Assay

CASE (0–100 *μ*g/mL) was diluted in phosphate buffer (10 mM, pH 7.4). Each sample was combined with 6 vol. of fluorescein (10 nM) and aliquoted into black 96-well plates and incubated for 30 min at 37°C without shaking. Following incubation, fluorescence was measured (Ex. 485 nm, Em. 520 nm) every 90 sec for 3 cycles to determine the background signal. Twenty-five (25) *μ*L of 2,2′-azobis-2-methyl-propanimidamide dihydrochloride (AAPH) or phosphate buffer (for “fluorescein only” control) was then added, and fluorescence was measured every 90 sec for 90 min. The slope of CASE-treated samples was compared to a standard curve of hydroxy-2,5,7,8-tetramethylchroman-2-carboxylic acid (trolox, the positive control) and results are expressed as trolox equivalents.

### 2.4. Electron Paramagnetic Resonance Spectroscopy (EPR)

The radical scavenging activity of CASE was measured using electron paramagnetic resonance by the method of Voest, Faassen, & Marx, (1993) with some modifications [12]. The EPR spectra of 4-hydroxy-2,2,6,6-tetramethylpiperidinyloxy (TEMPOL) were recorded on a Bruker eScan R X-band spectrometer at 37°C. The EPR microwave power was set to 37.86 mW, the modulation frequency was 86 kHz, and a sweep time of 2.62 s was used. Each time point for a sample was scanned a total of 3 times. CASE was diluted in phosphate buffered saline (100 mM, pH 7.4) and combined with AAPH (40 mM) and 24 *μ*M TEMPOL. The kinetics of TEMPOL reduction was studied over 120 min. Trolox (120 *μ*M) was used as the positive control.

### 2.5. Prevention of Lipid Oxidation in Emulsion

A 5% corn oil-in-water emulsion in sodium phosphate buffer (pH 7.0) containing 1% tween 20 as an emulsifier and 0.02% sodium azide (as an antimicrobial) was prepared (mean particle size 0.19 ± 0.05 *μ*m), CASE was added, and the emulsions were incubated at 37°C for 62 days. Lipid hydroperoxides were measured using an established method with modifications [13]. In brief, emulsion samples were mixed with 5 vol. iso-octane/2-propanol (3:1 v/v) and vortexed and the organic phase was isolated by centrifugation. The organic phase was combined with 14 vol. methanol/*n*-butanol (2:1, v/v), 0.075 vol. ammonium thiocyanate (3.94 M), and 0.075 vol. ferrous iron solution (0.144 M FeSO_4_·7H_2_O in 0.132 M BaCl_2_). After 20 min, the absorbance of the solution was measured at 510 nm. Hydroperoxide concentrations were determined by comparison to a standard curve of cumene hydroperoxide.

### 2.6. Cell Culture

MCF7 human breast, H1299 human lung, LNCaP human prostate, and HT29 human colon cancer cell lines were purchased from American Type Culture Collection (Manassas, VA). H1299 and LNCaP cells were maintained in RPMI 1640 media supplemented with 10% fetal bovine serum, 100 U/mL penicillin, and 100 *μ*g/mL streptomycin. MCF7 cells were maintained under the same conditions with the addition of 1% sodium pyruvate to the medium. HT29 cells were maintained in McCoy's 5 A medium supplemented with 10% fetal bovine serum, 100 U/mL penicillin, and 100 *μ*g/mL streptomycin. Cell lines were maintained in log-phase growth at 37°C under a humidified 5% CO_2_ atmosphere.

### 2.7. Cell Viability Assay

The effect of CASE on cell viability was determined using the 3-(4,5-dimethylthiazol-2-yl)-2,5-diphenyltetrazolium bromide (MTT) assay. In brief, cells were seeded (10^4^ cells/well) in 96-well plates and allowed to attach overnight. Cells were treated with CASE in serum-complete medium at 37°C. After treatment, the cells were washed with CASE-free media and MTT was added to each well at a final concentration of 1 mg/mL. Conversion of the MTT dye to the formazan precipitate was determined spectrophotometrically at 540 nm. The viability of treated cells was normalized to medium-treated controls.

### 2.8. Cell Cycle Analysis

To determine the effect of CASE on cell cycle progression, LNCaP cells (1×10^6^) were plated in 75 cm^2^ flasks and allowed to attach for 36 h. The media was replaced with fresh serum-complete medium containing CASE and cells were treated for 12 h. Cells were harvested by trypsinization, washed with phosphate buffered saline (PBS), and fixed with 70% methanol. Cells were then treated with RNase (500 *μ*g/mL) and stained with propidium iodide (40 *μ*g/mL) for 30 min at 37°C. The cells were analyzed by flow cytometry using a Coulter Epics XL-MCL (Beckman Coulter, USA).

### 2.9. Apoptosis Analysis: Externalization of Phosphatidylserine

Externalization of phosphotidylserine was used as a marker of apoptosis. LNCaP cells (1×10^6^) were plated in 75 cm^2^ flasks and allowed to attach for 36 h. The media was replaced with the serum-complete medium containing CASE and incubated for 12 h. Cells were then costained with Annexin V-FITC and propidium iodide and harvested according to the manufacturer's instructions (ApoDETECT Annexin V-FITC Kit, Invitrogen, Fredrick, MD). The cells were analyzed by flow cytometry using a Coulter Epics XL-MCL (Beckman Coulter, USA).

### 2.10. Western Blot Analysis

LNCaP cells (1X10^6^) were seeded in 75 cm^2^ flasks and allowed to attach for 36 h. The media was replaced with media containing CASE at the IC_50_. To prepare total cell protein samples, the cells were washed with PBS, scraped off, and centrifuged at 1200 × g. Cell pellet was combined with lysis buffer (25 mM 3-(N-morpholino) propanesulfonic acid, 2 mM ethylenediaminetetraacetic acid (EDTA), 10% glycerol, 0.5% Nonidet P-40, and 0.02% sodium azide) containing 1:100 phosphatase inhibitor I, phosphatase inhibitor II, and protease inhibitor. The samples were mixed and disrupted by freeze thawing.

To prepare nuclear extracts, cells were treated as above and were scraped and centrifuged at 800 × g for 10 min at 4°C. The cells were suspended in buffer A (10 mM 4-(2-hydroxyethyl)-1-piperazineethanesulfonic acid (HEPES), pH 7.9, 1.5 mM MgCl_2_, 10 mM KCl, 0.5 mM dithiothreitol (DTT), and 0.1% Nonidet P40). The samples were incubated in ice for 10 min and centrifuged at 12000 × g for 2 min at 4°C. The pellet was resuspended in buffer B (10 mM HEPES, pH 7.9, 1.5 mM MgCl_2_, 0.4 M NaCl, 0.5 mM DTT. 0.2 mM EDTA, 0.5 mM phenylmethylsulfonyl fluoride (PMSF), and 25% glycerol). The tubes were then vortexed and incubated on ice for 15 min with mixing every 5 min. They were then centrifuged at 10,000 × g for 10 min at 4°C. The supernatant was removed and used as nuclear fraction.

For both nuclear and whole cell lysates, protein (60 *μ*g of whole cell protein or 20 *μ*g for nuclear extract) was combined with an equal volume of loading buffer and resolved by SDS-polyacrylamide gel electrophoresis. Samples were transferred to nitrocellulose membranes, blocked for 1 h with blocking buffer (Li-Cor Biosciences), and probed with primary antibody overnight at 4°C. The bands were visualized after incubation with a fluorescent-conjugated secondary antibody (Li-Cor Biosciences) using an Odyssey Infrared scanning system (Li-Cor Biosciences). Total cellular protein expression was normalized to *β*-actin, whereas nuclear protein expression was normalized to histone H3 expression.

### 2.11. Statistical Analysis

Data are presented as the mean ± SD unless specified otherwise. GraphPad Prism (La Jolla, CA) was used for statistical analysis. Statistical differences were estimated by one way analysis of variance (ANOVA) with by Dunnett's post-test. P < 0.05 was considered statistically significant.

## 3. Results and Discussion

We have previously reported that CASE contains high levels of polyphenols and in the current study we explored the potential antioxidant and anticancer activity of the extract using* in vitro *models (Dabas et al., 2011).

**Figure 1:**
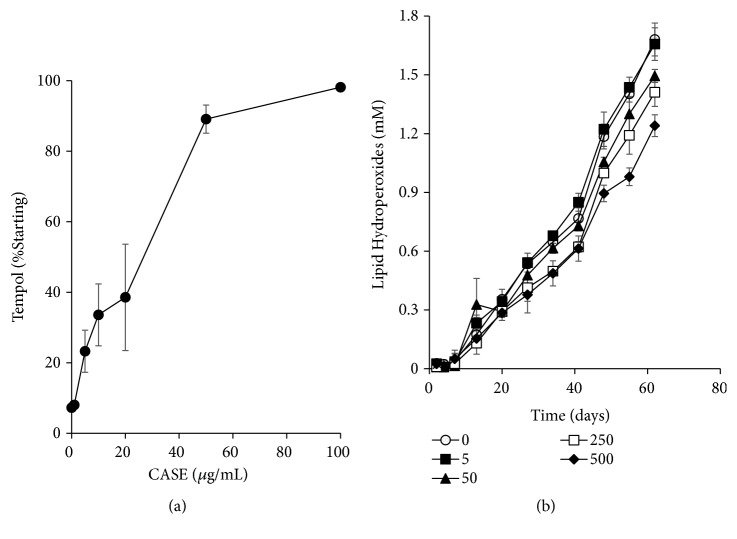
The radical scavenging and antioxidant activity of CASE. (a) Radical scavenging activity of CASE was measured using EPR. TEMPOL absorbance intensity was determined after incubation for 120 min at 37°C. The TEMPOL absorbance intensities were normalized to the intensity of control incubated with 120 *μ*M Trolox. (b) The antioxidant activity of CASE was determined in a corn oil-in-water emulsion. Different CASE concentrations up to 500 *μ*g/mL were used. Emulsions were incubated at 37°C for up to 62 days. Results are mean ±SEM of three independent experiments.

CASE demonstrated an ORAC value of 2012 ± 300 TE/mg. CASE (reconstituted in PBS, pH 7.4) displayed dose-dependent radical scavenging activity in the EPR assay with a 50% maximal effective concentration (EC_50_) = 42.1 *μ*g/mL (Figure 1(a)). CASE exhibited dose-dependent antioxidant activity in a model oil-in-water emulsion (Figure 1(b)). After 62 d, emulsions containing 500 *μ*g/mL CASE had 33% lower lipid hydroperoxide levels than vehicle-treated controls. The antioxidant activity of seeds has been previously correlated to the phenolic content [4–6]. For example, an acetone extract of the seed was compared to the same extract of the pulp. The seeds, which had 10-fold greater phenol content, had an ORAC value of 428.8 TE/g fresh weight compared to 11.6 TE/g in the pulp [5]. Hydroethanolic extracts of peels had higher total phenolic content (227.9 mg/g) compared to seeds; in the DPPH scavenging assay, seeds had a lower antioxidant potential (EC_50_ = 220 *μ*g/mL) compared to peels (EC_50_ = 149 *μ*g/mL) [6].

**Figure 2:**
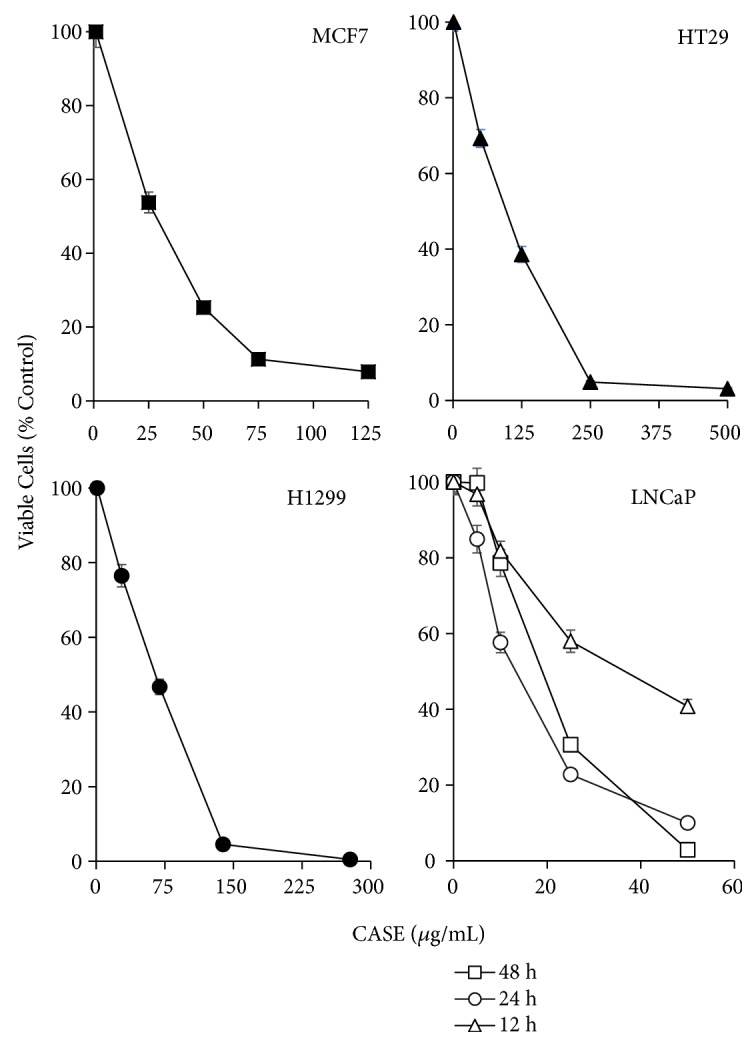
Effect of CASE treatment on the viability of selected human cancer cell lines in culture. MCF7, HT29, H1299, and LNCaP cells were treated for 48 h and cell viability was assessed using the MTT assay and normalized to vehicle-treated control cells. LNCaP cells were also treated for 12 and 24 h to determine the time-dependence of the effects on cell viability. Data represent the mean ±SEM of three independent experiments.

CASE dose-dependently reduced the viability of four human cancer cell lines (Figures 2(a)–2(d)). CASE inhibited the growth of LNCaP cells in a time-dependent manner. After 12, 24, and 48h, the IC_50_ = 42, 15, and 19 *μ*g/mL were obtained. Inhibitory effects against MCF7, HT29, and H1299 cells after 48 h treatment were also examined: the IC_50_ values were 19.1, 67.6, and 132.2 *μ*g/mL for MCF7, HT29, and H1299 cells, respectively.

**Figure 3:**
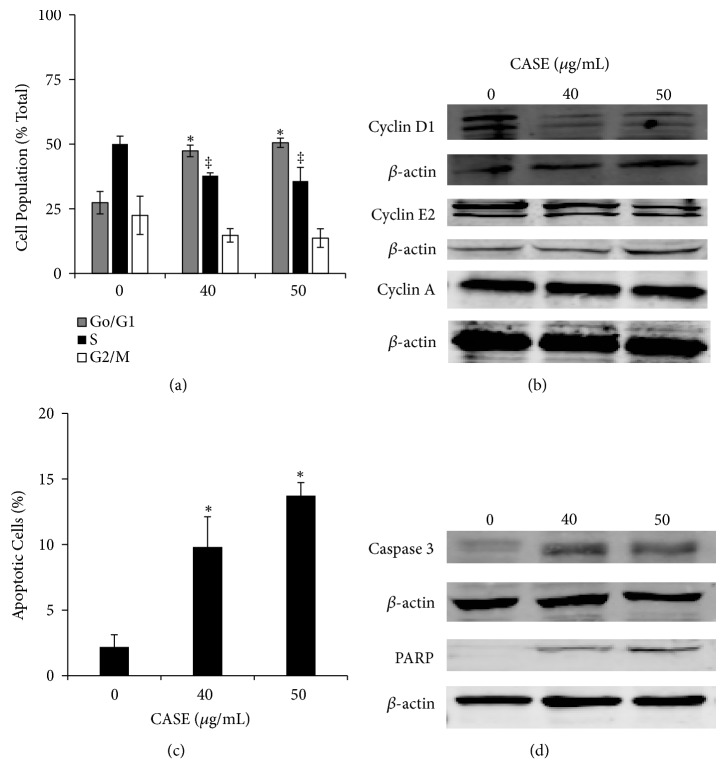
Induction of cell cycle arrest and apoptosis in LNCaP cells by CASE treatment. Cells were treated with CASE for 12 h. (a) Cells were fixed in methanol and stained with propidium iodide, and cell cycle analysis was performed using flow cytometry. (b) After 12 h treatment, the effect of CASE on the expression of cyclins was determined by western blot. Cyclin expression was normalized to expression of *β*-actin. Following treatment with CASE for 12 h, (c) induction of apoptosis was determined by quantifying externalization of phosphatidylserine using flow cytometry and (d) determining the extent of caspase 3 and PARP cleavage using western blot. Cleaved caspase-3 and PARP levels were normalized to *β*-actin. The blots shown are representative of three independent experiments. The bar graphs represent the mean of three independent experiments ±SEM.

CASE induced G_0_/G_1_ phase cell cycle arrest in LNCaP cells after 12 h treatment (Figure 3(a)). Treatment with CASE at the IC_50_ and IC_60_ increased the population of cells in the G_0_/G_1_ phase by 70 and 84%, respectively. A concomitant reduction in cells going to S and G_2_ phase was observed. Western blot analysis showed that the protein levels of both cyclin D_1_ and cyclin E_2_ were reduced by treatment with CASE, whereas no change in the expression of cyclin A was observed (Figure 3(b)). Investigation by flow cytometry revealed that treatment of LNCaP cells with CASE for 12 h induced externalization of phosphatidylserine by 5–7-fold (Figure 3(c)). CASE treatment also increased caspase 3 and PARP cleavage compared to control cells (Figure 3(d)).

Previously, Lee* et al*. (2008) reported similar observations following treatment of MDA-MB-231 breast cancer cells with an uncolored methanolic extract of avocado seed [7]. Treatment with 200 *μ*g/mL increased cleavage of caspase-3 and PARP. The differences in effective concentrations used in our study and the others may be related to differences in extract composition or use of different cells [7–9]. This difference requires further study.

**Figure 4:**
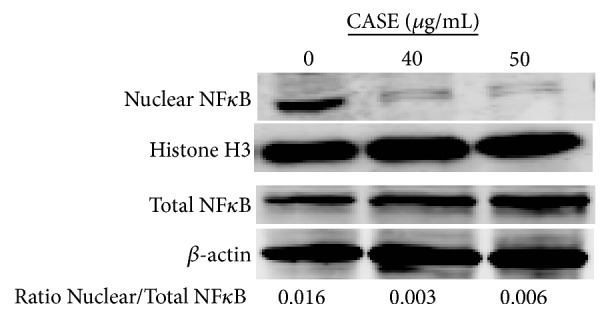
Effect of CASE on the expression of NF*κ*B in LNCaP cells. Western blot analysis was performed after 12 h treatment with CASE. Expression of total NF*κ*B was normalized to expression of *β*-actin. Nuclear levels of NF*κ*B were normalized to histone H3. Results are representative of three independent experiments.

Treatment with CASE reduced NFkB nuclear translocation by greater than 50%. By contrast, no significant effect of CASE was observed on the levels of total NF*κ*B in treated LNCaP cells (Figure 4). Constitutive overexpression of NF*κ*B or enhanced translocation of NF*κ*B to the nucleus confers a prosurvival phenotype to cancer cells. Constitutive activation of NF*κ*B has been reported in several types of cancer including prostate cancer [14]. Inhibition of NF*κ*B-mediated signaling by other dietary polyphenols and polyphenol-rich extracts has been shown to induce apoptosis in prostate cancer cells [15].

The present study has several strengths. We examined a panel of cell lines representing several important cancer types. We utilized both dose- and time-response study designs to provide greater clarity on the antioxidant and anticancer efficacy and potency of CASE. Finally, we investigated important anticancer mechanisms to provide insight into how CASE exerts its effects.

The study also had several limitations that need to be addressed in subsequent studies. First, the identity of the active antioxidant and anticancer components in CASE is not known. Additional studies are needed to isolate and identify these components. Second, we conducted only* in vitro* studies. Future studies are needed to evaluate the anticancer and antioxidant efficacy of CASE in appropriate animal models.

## 4. Conclusion

In summary, we have previously reported that CASE may have direct applications in food systems as a natural colorant [1]. In the present study we have observed that this extract has considerable antioxidant and anticancer activity* in vitro. *Our present results suggest that CASE may have a role as a functional food ingredient or as a source of novel natural antioxidants and anticancer compounds. Further studies are warranted to determine the* in vivo *anticancer activity of CASE and to identify the upstream mechanistic targets.

## Data Availability

Data will be provided upon request submitted to the corresponding author.

## Abbreviations

CASE: Colored avocado seed extract
FITC: Fluorescein isothiocyanate
GAE: Gallic acid equivalents
IC_50_: Half-maximal inhibitory concentration
MTT: 3,[4, 5-Dimethylthiazol-2-yl]-2, 5-diphenyltetrazolium bromide
NF*κ*B: Nuclear factor *κ* B
ORAC: Oxygen radical absorbance capacity
PARP: Poly (ADP-ribose) polymerase
TEMPOL: 4-Hydroxy-2,2,6,6-tetramethylpiperidinyloxy radical.
